# Case report: Three novel variants on SLC25A13 in four infants with neonatal intrahepatic cholestasis caused by citrin deficiency

**DOI:** 10.3389/fped.2023.1103877

**Published:** 2023-03-29

**Authors:** Kena Wang, Biao Zou, Fan Chen, Jianling Zhang, Zhihua Huang, Sainan Shu

**Affiliations:** Department of Pediatrics, Tongji Hospital of Tongji Medical Collage, Huazhong University of Science and Technology, Wuhan, China

**Keywords:** citrin deficiency, NICCD, SLC25A13, novel variant, prognosis

## Abstract

**Background:**

Neonatal intrahepatic cholestasis caused by citrin deficiency (NICCD) is a common clinical phenotype of citrin deficiency in infants. Its phenotype is atypical, so genetic testing is quite necessary for the diagnosis.

**Case presentation:**

We report 4 patients with jaundice and low body weight. Furthermore, the biochemical examination of all showed abnormal liver function and metabolic changes. DNA samples of the patients were extracted and subjected to genetic screening. All candidate pathogenic variants were validated by Sanger sequencing, and CNVs were ascertained by qPCR. The genetic screening revealed 6 variants in 4 patients, and all patients carried compound heterozygous variants of SLC25A13. Importantly, 3 variants were newly discovered: a nonsense mutation in exon17 (c.1803C > G), a frameshift mutation in exon 11(c.1141delG) and a deletion of the whole exon11. Thus, four NICCD patients were clearly caused by variants of SLC25A13. Biochemical indicators of all patients gradually returned to normal after dietary adjustment.

**Conclusions:**

Our study clarified the genetic etiology of the four infants, expanded the variant spectrum of SLC25A13, and provided a basis for genetic counseling of the family. Early diagnosis and intervention should be given to patients with NICCD.

## Introduction

Citrin deficiency (CD) is an autosomal recessive disease caused by SLC25A13 gene mutation. It includes three age-dependent clinical phenotypes: neonatal intrahepatic cholestasis caused by citrin deficiency(NICCD), adult-onset type II citrullinemia (CTLN2) and failure to thrive and dyslipidemia caused by citrin deficiency (FTTDCD) between NICCD and CTLN2 stages (1). NICCD is one of the most common genetic metabolic diseases causing infantile cholestatic hepatopathy, which is associated with persistent jaundice, abnormal coagulation function, hepatosplenomegaly, chubby face, growth retardation, metabolic abnormalities, etc (2)., but these features are not pathognomonic. So genetic analysis is regarded as a reliable method for the definite diagnosis of NICCD. With the development of genetic testing, we recognize that although NICCD is a pan-ethnic disease, it is still prevalent in East Asia. Meanwhile, China is a high-incidence area, the mutation carrier rate of SLC25A13 gene is as high as 1/65. The type of variation of SLC25A13 is also different in different regions. In China, c.852_855del4, c.1638_1660dup, IVS6 + 5G > A, and IVS16ins3kb are the four most common mutations of SLC25A13.In this study, we performed genetic screening on 4 patients with jaundice and every patient carried compound heterozygous mutations with a common variant. Unexpectedly, we also detected 3 novel variants of SLC25A13, including a nonsense mutation, a frameshift mutation, and a whole exon deletion. These findings enriched the variant spectrum of NICCD and guided clinical diagnosis and genetic counseling.

## Subjects and methods

### Subjects

A total of 4 patients (3 females and 1 male) from non-consanguineous families were enrolled in this study. They were all full-term, and some patients (patient 1, 2 and 4) showed low birth weight and/or low birth length. They all appeared jaundiced with normal color stool about 3 days after birth. They were given blue light irradiation, ursodeoxycholic acid, liver protection drugs and other symptomatic treatment, but there was no significant improvement in their symptoms. Patients 1, 2, and 3 were admitted to our hospital for jaundice, and patient 4 for jaundice and abnormal liver function. All of them were underweight and had no signs of chubby faces. Yellow staining of the skin and sclera was observed. Their livers were palpable 2–3 cm under the rib cage. Laboratory tests are shown in Table 1. Laboratory tests in all patients showed transaminitis, hyperbilirubinemia, elevated GGT, and hypoalbuminemia. Some patients had hypofibrinogenemia accompanied by metabolic abnormalities: hyperlactatemia, hypoglycemia, hypertriglyceridemia, citrullinemia, and elevated pyruvate. Except for patient 1, who did not check for alpha-fetoprotein (AFP), the AFP of the remaining patients was extremely elevated. Ultrasonography in Patients 1 and 2 showed the features of fatty liver. Patient 4 had no sign of fatty liver on the ultrasound, however the liver biopsy showed hepatic steatosis.

**Table 1:** Clinical features of the 4 patients.

	Patient 1	Patient 2	Patient 3	Patient 4
Sex	Female	Female	Male	Female
Gestational age (week)	41	37	39^ + 6^	37^ + 5^
Birth weight (kg)	2.9 (3–10th)	2.8 (25–50th)	3.2 (25–50th)	2.17 (<3rd)
Birth height (cm)	50 (50–75th)	46 (3–10th)	50 (50–75th)	45 (<3rd)
Visiting ages	1 month 25 days	2 months 6 days	2 months 1 days	4 months 12 days
Hospital weight	4 kg (<3rd)	4 kg (<3rd)	4.9 kg (3–10th)	6 kg (<3rd)
ALT (≤41 U/L)	23	35	26	36
AST (≤40 U/L)	41	113	122	76
ALB (38–54 g/L)	27.9	27.3	31.2	37
TB (≤26 μmol/L)	89.7	209.7	136.3	51.9
DB (≤8 μmol/L)	41.1	105.8	57.6	44.2
GGT (10–71 U/L)	295	174	167	120
TBA (≤10 *μ*mol/L)	95.7	163.5	255.1	131.7
TC (<5.18 mmol/L)	5.08	3.89	4.5	6.05
TG (<1.7 mmol/L)	–	1.85	1.95	2.92
Fib (1.7–4.1 g/L)	1.71	0.68	0.94	2.57
BS (4.11–6.05 mmol/L)	2.49	5.7	2.16	5
Ammonia (16–60 μmol/L)	70	81	98	66
LA (0.5–2.2 mmol/L)	7.02	8.14	2.26	1.48
PA (20–100 μmol/L)	141.7	71.6	96	35.1
AFP (≤7 ng/ml)	–	>60,500	>60,500	>60,500
PT (11.5–14.0 s)	13.8	20.7	16.9	13.8
Hepatosplenic ultrasound	Fatty liver-like changes	Fatty liver-like changes, gallbladder wall is thickened and not smooth, modest splenomegaly	Modest hepatosplenomegaly	Modest hepatosplenomegaly

ALT, alanine aminotransferase; AST, aspartate aminotransferase; ALB, albumin; TB, total bilirubin; DB, direct bilirubin; GGT, gamma-glutamyltransferase; TBA, total bile acids; TC, total cholesterol; TG, triglyceride; Fib, fibrinogen; BS, blood sugar; LA, lactic acid; PA: pyruvic acid; AFP, alpha-fetoprotein; PT, prothrombin time.

### Genetic testing

Genomic DNA was extracted from the peripheral blood of all probands. Genetic screening was performed for each proband: proband 1(captured by exome kit from Agilent, sequenced on Illumina platform); proband 2 (captured by metabolic liver disease panel from MyGenostics, sequenced on DNBSEQ-T7); proband 3 (captured by a panel from amcarelab, sequenced on Illumina platform); proband 4 (captured by a panel from our in-house liver disease panel, sequenced on Illumina platform). All sequencing data were mapped with human reference genome(hg19), and variants were classified according to ACMG guidelines. All candidate pathogenic variants were validated by Sanger sequencing, and CNVs were validated by quantitative real-time polymerase chain reaction (qPCR).

## Results

### SLC25A13 mutations

**Figure 1:**
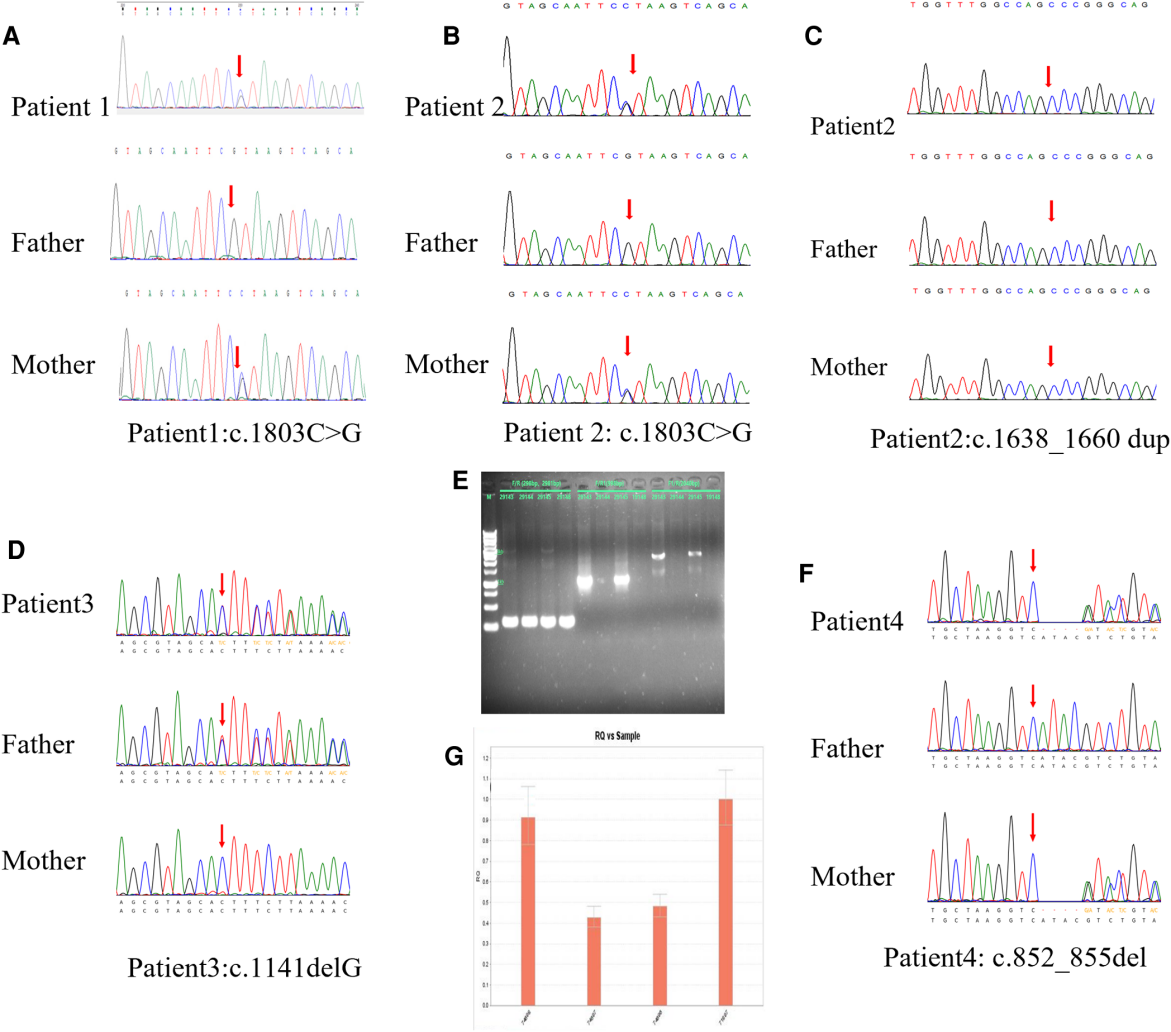
(**A**) Patient 1 carried the variant of c.1803C > G, the results of sanger sequencing indicated that it was inherited from her mother. (**B**)The novel variant c.1803C > G was detected in patient 2, which is derived from her mother. (**C**) c.1663_1664insGAGATTACAGGTGGCTGCCCGGG in patient 2 was confirmed from her father by Sanger sequencing. (**D**)The deletion variant c.1141delG in patient 3 was from his father. (**E**) Patient 3 and his mother is heterozygous nonsense mutation of c.1750_1751 ins3Kb, his father is normal. (**F**) Patient 4 was a carrier of c.852_855del, which is derived from her mother. (**G**) The another variant exon11 del in patient 4 were validated by qPCR, the image from left to right is her mother, her father, herself and the sample, so that the mutation originated from her father.

Compound heterozygous variants from SLAC25A13 were detected in all four probands; all variants were classified as likely pathogenic or pathogenic according to ACMG guidelines (3) (Table 2): Patient 1 [NM001160210: c.1803C > G, p.Y601X (Figure 1A) and IVS16ins3kb, p.A584fs]; Patient 2 [NM001160210: c.1803C > G, p.Y601X (Figure 1B) and c.1663_1664insGAGATTACAGGTGGCTGCCCGGG, p.A555fs] (Figure 1C) ]; Patient 3 [NM_014251: c.1141delG, p.V381Cfs*27 (Figure 1D) and c.1750_1751 ins3Kb (Figure 1E), patient 4 [NM_014251: c.852_855del, p.M285Pfs*2 (Figure 1F) and exon11 del (Figure 1G)].

**Table 2:** Variants detected summary.

Nucleotide change	c.1803C > G	IVS16ins3kb	c.1663_1664insGAGATTACAGGTGGCTGCCCGGG	c.1141delG	c.852_855del	exon11del
Chromosome location	chr7: 95751008	chr7: 95751062–95751078	chr7: 95751240–95751241	chr7: 95813625	chr7: 95818684	chr7: 95813589-95813747
Proband	1 and 2	1 and 3	2	3	4	4
Exon/intron	exon17	intron16	exon16	exon11	exon9	Exon11
Amino acid change	p.Y601X	–	p.A555fs	p.V381Cfs*27	p.M285Pfs*2	–
ACMG	LP	P	P	LP	P	LP
Reported	No	Yes	Yes	No	Yes	No

### Clinical outcome

All four patients adjusted their diets: breastfeeding was stopped and it was replaced by a lactose-free and MCT-rich formula. The clinical manifestations improved significantly and none of them suffered long-term complications (We called the family of the patient 2, who informed us that the patient's liver function and AFP levels had returned to normal, but the report was lost. The follow-up results of patients 1, 3, and 4 are showed in Table 3). Most of the patients revealed specific preference for high-protein food but not with an obvious aversion to foods high in carbohydrate.

**Table 3:** Follow-up of the patients.

	Patient 1	Patient 2	Patient 3	Patient 4
Age	6Y1M	7Y7M	3Y8M	2Y3M
Weight (kg)	19.75 (25–50th)	125 (25–50th)	16 (50–75th)	12.5 (25–50th)
Height (cm)	119 (50–75th)	26 (50–75th)	101 (50–75th)	89 (25–50th)
ALT (≤U/L)	11	N	12	18
AST (≤40 U/L)	24	N	49	37
ALB (38–54 g/L)	44.7	N	46	49.5
TB (≤26 µmol/L)	10.4	N	13.2	9.3
DB (≤8 µmol/L)	4.1	N	3.5	4.4
GGT (10–71 U/L)	15	N	12	16
TBA (≤10 µmol/L)	1.4	N	5	24.8
TC (<5.18 mmol/L)	4.76	N	2.59	3.99
TG (<1.7 mmol/L)	0.66	N	1.16	0.67
AFP (≤7 ng/mL)	3.42	N	2.12	0.93
Complication	No	No	No	No
Dietary preferences	Like protein	Like protein	Like protein	No specific food preferences

N, normal.

## Discussion

SLC25A13 is the pathogenic gene of NICCD, which encodes the citrin protein. Citrin plays significant roles in the urea cycle, malic acid-aspartate shuttle, and gluconeogenesis (3). As a result, variants of SLC25A13 can lead to metabolic abnormalities such as citrullinemia, hyperammonemia, hypoglycemia, and hyperlipidemia. Our study results were similar to those of the previous studies (4), some patients with NICCD showed low birth weight and low birth height according to sex and gestational age. At the same time, the patients with NICCD can also be accompanied by elevated liver enzymes, hyperbilirubinemia, significantly elevated alpha-fetoprotein, hypoalbuminemia, prolonged prothrombin time and fatty liver. Predictably, four patients in our study also had these characteristics. The sign of hepatic steatosis is common in patients with NICCD. While both the ultrasonography and liver biopsy of patient 3 did not indicate evidence of hepatic steatosis. Therefore, consistent with Miyamoto's report (5), NICCD cannot be ruled out even if hepatic steatosis is not seen in patients with cholestasis. None of the above changes are specific. A variety of neonatal intrahepatic cholestasis (such as cytomegalovirus hepatitis, Alagille syndrome, etc.) can manifest as jaundice, elevated liver enzyme, hepatosplenomegaly, and growth retardation. Meanwhile, other inherited metabolic diseases(such as galactosemia (6), hepatocerebral mitochondrial DNA depletion syndrome (7–9), arginylsuccinuria (10), etc.) can also have symptoms similar to NICCD. It can be seen that it is a big challenge to distinguish NICCD from other diseases. Therefore, we should actively conduct genetic testing for patients with unexplained infantile cholestasis to avoid misdiagnosis and missed diagnosis.

So far, more than 300 variants of SLC25A13 have been reported worldwide, including 292 single nucleotide variations, 28 deletion mutations, 8 duplation mutations, 4 microsatellite mutations, 3 insertion mutations, and 1 insertional deletion variant. In our study, four patients all harbored compound heterozygous variants of SLC25A13, and a total of 6 variant types were detected, among which three have not been reported: c.1803C > G, c.1141delG and exon 11 del. The common mutation c.1803C > G identified in patient1 and 2, which is located on exon 17 and is predicted to cause the early appearance of amino acid termination code (tyrosine > termination) in protein synthesis, and according to ACMG guidelines, pathogenicity analysis is PVS1 + PM2 + PP4, it is classified as likely pathogenic, so far as we know, which has not been reported previously. While in our study, it was found in two patients and two family members. After a detailed medical history we found no consanguinity between the two families; however, they all came from Hubei Province, China, which spans the Yangtze River and belongs to the central city in China. The previous large-scale studies on NICCD were generally in the south and north of China, with few central cities. As we all know, there is a significant difference in the mutation carrier rate of SLC25A13 gene between North and South populations along the boundary of 30° North latitude in China. The results of our study suggested that the carrier rates of c.1803C > G may be frequent in the central cities of China, and there are differences in the distribution of the mutation spectrum in different geographical areas. As for the relationship between genotype and phenotype, although some studies declared that there were likely to be phenotype–genotype correlations in the specific genotypes (11), in our study, no obvious genotype-phenotype relationship seems to be found. For example, heterozygous genotypes including c.1803C > G were found in patient 1 and 2, but their birth weight, AST, Fib, PA and prothrombin time have a marked difference. And patient 1 and 3 carried the heterozygous variants including IVS16ins3kb, but they didn't present the same clinical manifestations. So we do not hold that there is a significant genotype-phenotype relationship between patients with NICCD. The number of cases in our study was small, so further studies with more samples may be required. c.1141delG is a novel code-shifted mutation that has not been reported in relevant clinical cases and our reference population gene database. Moreover, it is predicted that it may lead to the early occurrence of amino acid termination ciphers in protein synthesis. According to the ACMG guidelines, the pathogenicity analysis is PVS1 + PM2 + PP4, so it is likely pathogenic. Exon deletion is a rare variant type among SLC25A13 variants, which are mainly caused by large fragment deletion, including exon deletion, such as c.329–154_468 + 2352del2646bp (deletion of exon 5) (12), c.70–862_c.212 + 3527 del4532bp (deletion of exon 3) (13), Ex16 + 74_IVS17-32del516 (deletion of exon 17) (14), c.329–1687 -c.468 + 3865del5692bp (deletion of exon 5) (15), c.1019_1177 + 893del (deletion of exon 11) (16), c.1312–2860_1452 + 988del (deletion of exon 14) (17), c.1312–4144_1452 + 3373del (deletion of exon 14) (17) and c.3251 _c.15 + 18443del21709bp (deletion of exon 1) (18). Partial abnormal splicing of partially intron regions leads to exome deletions in mRNA, such as IVS4ins6 kb (skipping of exon 5) (19), IVS11+1 G > A (skipping of exon 11) (20), IVS15 + 1G > T (skipping of exon 15) (21) and IVS13+1G > A (skipping of exon 13) (22). In this study, after high-throughput sequencing analysis, patient 4 was found to carry a common variant c.852_855del and be accompanied by copy number variation. In order to clarify the definite genetic etiology of the patient, qPCR verification was performed. Eventually, another variant (exon 11 del) was found in the patient. So, this patient was confirmed as NICCD caused by compound heterozygous variants of SLC25A13. The other three variants identified in this study: c.1663_1664insGAGATTACAGGTGGCTGCCCGGG (12, 23), IVS16ins3kb (15, 24–27), and c.852_855del (23, 24, 28–31) have been reported in multiple literatures and are three of the most common variants of SLC25A13 in China.

Conventional genetic screening, such as whole exon sequencing, can only detect point mutations or small deletions. However, it was found in a study by Tokuhara (32) that about 15% of the complex heterozygotes or homozygotes of the SLC25A13 gene could not be identified by the routine methods. Fortunately, the emergence of multiplex ligation-dependent probe amplification (MLPA) (20), Southern blots restriction fragment analysis (33), custom fluorescence *in situ* hybridization (FISH) (34), and qPCR (21) can help us better detect this part of the variants. Therefore, additional testing is recommended for the patients who are clinically considered for NICCD but have only one heterozygous mutation detected by conventional genetic screening.

After the dietary adjustment, the liver function such as ALT, GGT, TB, DB, TBA, ALB, AFP returned to normal at the last review (Table 3). The patients were all underweight at the time of their first visit to our hospital, although the follow-up time of our study was short (the maximum is approximately seven years), the height and weight of them gradually returned to normal. Recently there were cases of liver cancer (35) in NICCD patients have been reported, it suggested that NICCD is not always harmless. Fortunately, none of the long-term complications including liver cirrhosis, hepatoadenoma or hepatocellular carcinoma, pancreatitis and so on occurred in the patients in this study. A recent study by Kido (11) found that the height and the weight of patients with NICCD were high than those with CTLN2, in the meantime long-term complications were seen in patients with CTLN2 but not in older NICCD patients. It is speculated that medical management including dietary interventions can help patients. Therefore, we emphasize here the necessity for dietary intervention in patients with NICCD to alleviate clinical manifestations and prevent long-term complications.

In summary, our study reviewed the clinical manifestations, genetic test results, and prognosis of NICCD patients from 4 unrelated families. There are 6 variants of SLC25A13 were identified, and three novel variants were found: c.1803C > G, c.1141delG, and exon 11 del. This result clarified their genetic etiology and provided a strong basis for the clinical diagnosis of patients. At the same time, the detection of the novel variants enriched the variant spectrum of the SLC25A13, which further laid the foundation for the correlation study of the genotype and phenotype of NICCD.

## Data Availability

The Genetic screening data presented in the study are deposited in the Genome Sequence Archive or Human (https://ngdc.cncb.ac.cn/gsa-human/), with accession number HRA003718.
